# The impact of scars on health-related quality of life after breast surgery: a qualitative exploration

**DOI:** 10.1007/s11764-020-00926-3

**Published:** 2020-08-20

**Authors:** Kristel E. Everaars, Marlies Welbie, Stefan Hummelink, Esther P. M. Tjin, Erik H. de Laat, Dietmar J. O. Ulrich

**Affiliations:** 1 Department of Plastic Surgery, Radboudumc, Geert Grooteplein-Zuid 10, 6500 HB Nijmegen, The Netherlands; 2 Research Center Healthy and Sustainable Living, Research group Innovation in Healthcare Processes in Pharmacology, University of Applied Sciences Utrecht, Heidelberglaan 7, 3584 CS Utrecht, The Netherlands; 3 Research Center Healthy and Sustainable Living, Research group Methodology of Practice-Based Research, University of Applied Sciences Utrecht, Heidelberglaan 7, 3584 CS Utrecht, The Netherlands

**Keywords:** Scars, Breast cancer, Surgery, Satisfaction, Health related quality of life, Qualitative study

## Abstract

**Purpose:**

The purpose of this research was to explore women’s experiences after breast surgery with scar characteristics and symptoms, and its impact on their health-related quality of life (HRQOL).

**Material and methods:**

A qualitative study using semi-structured face-to-face interviews was conducted among women following prophylactic, oncologic, or reconstructive breast surgery in the Netherlands. A directed content analysis was performed using guiding themes. Themes were “physical and sensory symptoms,” “impact of scar symptoms,” “personal factors,” “impact of scar interventions,” and “change over time.”

**Results:**

The study population consisted of 26 women after breast surgery. Women experienced a wide range of symptoms like adherence, stiffness, pain, and uncomfortable sensations. Scar characteristics as visibility, location, texture, and size, influenced satisfaction with their appearance. The impact of scar symptoms is reflected in physical, social, emotional, and cognitive functioning, thereby affecting HRQOL. The experienced impact on HRQOL depended on several factors, like personal factors as the degree of acceptance and environmental factors like social support.

**Conclusion:**

Women can experience a diversity of scar characteristics and symptoms, which play a central role in the perceived impact on HRQOL. Since scarring can have a considerable impact on HRQOL, scarring after prophylactic, oncologic and reconstructive breast surgery should be given more attention in clinical practice and research.

**Implications for Cancer Survivors:**

Considering scarring as a common late effect after breast surgery and understanding the variety of experiences, which could impact HRQOL of women, can be beneficial in sufficient information provision, expectation management, and informed decision making.

## Introduction

Breast cancer is the most common cancer among women worldwide, with 1.38 million newly diagnosed cases each year [1]. About 80% of these women need surgery [2]. Regardless of the type of surgery, all forms of surgery result in scarring, which can have a strong impact on quality of life (QOL) [3, 4]. Scars in general can have a profound impact on physical, social, emotional [3, 5, 6], and cognitive functioning, affecting health-related quality of life (HRQOL) [5, 6]. Because of improved survival rates, more knowledge about late effects after oncologic breast surgery [7] such as scarring becomes highly relevant.

After oncologic breast surgery, 18% of the women experience their breast scars worse than expected [8], and about 10–30% is dissatisfied with the appearance of their scar [9, 10]. Although breast reconstruction surgery provides the opportunity to restore the appearance of the breast(s), scarring can be a significant post-operative concern and a major source of dissatisfaction with the overall surgical outcome [11]. Recently, it has been reported that breast scarring after reconstruction explained 11% of the variance in QOL, 8% of the variance of breast appearance, and 6% of the outcome satisfaction [12].

Although these findings show that breast scar–related complaints may be a common late effect after breast surgery, little is known about which scar characteristics and symptoms affect breast scar dissatisfaction or complaints. Insights into breast scarring are limited [11], although there are indications that women can experience symptoms like pain and itching. Thereby, satisfaction can be affected by scar characteristics such as difference in color, thickness, stiffness, and irregularity compared with normal skin [13].

Since insights regarding the impact of breast scars are based on a single item question about scarring [8–10] or a total satisfaction score [12], the patient’s perspective is important to take into consideration in order to gain an understanding into the diversity of scar characteristics, symptoms, and how women feel and function with their breast scars.

In order to explore women’s experiences with scar characteristics and symptoms after prophylactic, oncologic and reconstructive breast surgery, and its impact on their HRQOL, a qualitative study was conducted.

## Material and methods

### Design

This study employed a qualitative descriptive design including semi-structured interviews among women following prophylactic, oncologic, or reconstructive breast surgery. A directed content analysis [14] was performed using guiding themes. The study was approved by the Medical Ethics Committee of the Radboudumc Nijmegen The Netherlands.

### Sampling strategy

In order to gain insight into a variety of experiences with scarring after breast surgery, we included a heterogeneous sample of women with a diversity in types of breast surgery: lumpectomy and mastectomy (prophylactic and curative) with or without breast reconstruction (for full details see Table 1). Participants were randomly recruited by their dermal therapist in primary care (where they were treated for ongoing side effects after breast surgery, mainly lymphedema). Women were recruited regardless the presence, degree, or type of scar symptoms. Other recruitment selection criteria were various types of oncologic or prophylactic breast surgery, over 18 years old, spoke the Dutch language, and were able to understand and sign the informed consent. Any chemotherapy and/or radiation should have been completed. After inclusion (and preliminary analysis) of 21 participants from primary care, subsequently five women (without a predetermined request for help for side effects) after breast reconstruction were recruited, in order to achieve a heterogeneous diverse sample in surgical background. Recruitment was done by the first author (K.E.) from the breast reconstruction database of Radboudumc. During the iterative process of analysis and enrolment of participants, themes were checked for data saturation; this was considered to be reached when both deductively and inductively no new themes were obtained from the interviews [15]. During the interviews with the women who had undergone breast reconstruction, no new themes emerged and data saturation was considered to be reached.

**Table 1:** Individual patient characteristics

Pseudonym (age)	Type of surgery	SN	ALND	RT	CT
Emily (32)*	2015: bilateral mastectomy (one prophylactic) 2017: delayed implants, 1 failed	x		x	x
Patty (43)	2017: lumpectomy			x	
Violet (44)	2015: bilateral mastectomy, delayed implant reconstruction		x	x	x
Maggy (45)*	2009: unilateral mastectomy, 2017: prophylactic unilateral mastectomy, delayed DIEP flap		x		x
Susan (47)	2016: prophylactic bilateral mastectomy + immediate implants, both failed				
Liz (49)	2010: bilateral mastectomy (one prophylactic), 2012: delayed DIEP flap				x
Grace (50)	2012: unilateral mastectomy		x	x	x
Jessica (51)*	2013: prophylactic bilateral mastectomy, failed implants, 2014: DIEP flap				
Marianne (52)	2004: unilateral mastectomy		x	x	x
Audrey (53)	2013: lumpectomy	x		x	x
Evelyn (53)*	2015: unilateral mastectomy, 2017: LD flap + implant + nipple	x		x	x
Stacey (54)	1989: unilateral mastectomy, 1998: delayed implant reconstruction		x	x	x
Jill (58)	2009: lumpectomy	x		x	
Norah (59)	2004: unilateral mastectomy				
Rosie (59)	2014: bilateral mastectomy	x	x	x	x
Lisa (61)	2017: lumpectomy	x		x	
Hannah (62)	2015: bilateral mastectomy (one prophylactic), implant reconstruction + nipple	x		x	x
Lily (63)*	2004: lumpectomy, LD flap, 2014: unilateral mastectomy, 2016: PAP flap + nipple		x	x	x
Lucy (63)	2017: lumpectomy			x	
Leah (65)	1995: unilateral mastectomy		x	x	x
Margaret (65)	2016: lumpectomy	x		x	
Emma (66)	2006: lumpectomy, 2014: bilateral mastectomy (one prophylactic)		x	x	x
Katherine (66)	2017: lumpectomy			x	x
Suzy (66)	2004: lumpectomy		x	x	x
Charlotte (76)	1994: lumpectomy, 2017: unilateral mastectomy		x	x	x
Betty (77)	1980: unilateral mastectomy, 1982: tumor excision + delayed LD flap		x	x	

The participants’ names are replaced by pseudonyms and are arranged by the women’s age. Women represented with asterisk were recruited from Radboudumc

*SN* sentinel node, *ALND* axillary lymph node dissection, *RT* radiotherapy, *CT* chemotherapy

### Data collection

Face-to-face, semi-structured interviews were held by the first author (K.E.) on a location determined by the participant’s preference. We used a pilot tested interview guide, structured by the following five themes: “physical and sensory symptoms,” “impact of scar symptoms,” “personal factors,” “impact of scar interventions,” and “change over time.” These themes were derived from the conceptual HRQOL burn scar model of Simons et al. [6], which was based on existing HRQOL models [6]. These common themes in HRQOL [16] were also identified in research after breast oncology [17].

Interviews with a duration between 24 and 90 min were conducted and recorded in the period of October 2017 until April 2018. Transcripts and other sensitive data were stored at the Digital Research Environment of Radboudumc.

### Data analysis

In accordance with a directed content analysis, our systematic analysis began during the early stages of data collection using guiding themes [14]. After each interview was conducted, it was summarized by filling in the five themes of Simons [6]. It was then transcribed verbatim, using a transcription protocol. Recruiting, interviewing, and data analyzing were an iterative process.

The five themes with corresponding codes [6] were applied deductively and formed the initial coding list. To ensure consistency we formulated definitions for the themes, generated code rules, and anchor codes for each code, which together formed a code manual. The appendix depicts a summary of the code manual. During the early stages, the first three interviews were coded by two researchers (K.E., M.W.) and two research assistants in order to check our code manual and to improve the trustworthiness and the inter-coder reliability. During the iterative process of coding, revising, and discussing, the initial codes where refined and complemented with inductively formulated codes on the basis of empirical data in perspective to the research objective. In a later stage, all transcripts were double checked independently by K.E. and two research assistants to ensure consistency of code assignment. The patient’s names were replaced by pseudonyms. ATLAS.ti version 8.3.20.0 (Scientific Software Development GmbH, Berlin, Germany) was used for qualitative data management.

## Results

### Patient characteristics

Data saturation was obtained after interviews with 26 participants, 21 women were recruited by their dermal therapist, and additional five women who underwent a breast reconstruction were recruited from Radboudumc (see Table 1). The average age of the participants was 56.9 (± 10.4) and varied between 32 and 77 years old. On average, the last surgery was 6.5 (± 8.4) years ago. Women had undergone a variety, and often a combination of surgical interventions, see Table 1 for individual patient characteristics.

### The exploration of themes

#### Theme 1: Physical and sensory symptoms

Most women mentioned both positive and negative physical scar characteristics when asked to describe their scars. Characteristics that determined the degree of satisfaction with the scar appearance were color, width, thickness, location, visibility, the size of the scar, and dog-ears. A recurring topic of conversation was the changed appearance of their breast(s) caused by scarring.

Emma: “But, that it would look like this, that I didn’t fully realize.” “Ok, and what does it look like?” **“**Well that the scars run from here to there.”

Some women even felt mutilated, in order to describe their breast scars women used phrases such as “patchwork,” “war zone,” “zipper,” “roulade,” “face of a bulldog,” “monkey,” or “fat worm.”

Betty: “And why do you feel mutilated by those scars? Can you tell me something about that?”

“The operation of this nipple didn’t succeed, that’s such a big piece…That breast is completely disfigured. And the other side… I can live with that somehow, there I have all kinds of scars, but the real breast is still there. So well, it is only scar tissue you see.”

In contrast, some others were satisfied with the appearance of their scar(s).

Marianne: “It’s a pretty fine line and honestly not that much trouble […] it healed very nicely, the skin has tightened and it all looks neat and good.”

Besides the particular visible characteristics, more than half of the women mentioned physical scar symptoms like tightness, adherence, retraction, and stiffness, also affecting appearance but moreover often impacting the range of motion of the arm. Scar pain was the most common sensory symptom experienced by eighteen participants, ranging from “stabbing,” “sharp,” and “nagging” pain, followed and often in combination with tight sensations. For some women, pain was accompanied by cramps and the feeling of tearing of the scar tissue.

Liz: “If you do something, or fall, you will get such a cramp. You feel like it might come loose [..] So it’s such a tight feeling, that when you make an abrupt movement…for example, when I walk the dog and he encounters another dog and he starts pulling, then it’s like you’re pulled apart, or that it springs open.”

Furthermore, participants described altered sensations, about half of the women experienced numbness and uncomfortable sensations (stinging, tingling), and a few experienced hypersensitivity or itching.

#### Theme 2: Impact of scar symptoms

The impact of scarring on HRQOL is divided in subthemes: physical, emotional, social, and cognitive functioning and the impact of the environment on scar symptoms.

#### *Physical functioning*

The impact of symptoms, especially pain and scar adherence, were mentioned by more than half of the women, saying that it affects their arm movement. For a majority of the women this impacts their daily life in areas like sleep, leisure, housekeeping, work and sports.

Leah: “Really, to just reach out like that is very difficult and very painful and it doesn’t go so well either. So in everyday life you come across a whole lot of things, in which you can actually not use that left arm in the same way as the right one.”

Some other women, who did experience symptoms, did not feel impacted in physical function, they usually felt uncomfortable sensations or itching, but this was also mentioned for pain and pulling sensations.

Lily: “Do you feel any inconvenience?” “No! no.. You see, if it doesn’t work with the one arm, then I will do it with the other arm!”

#### *Emotional functioning*

Feelings of loss, sadness, and anger were mentioned with regard to the limitations in physical functioning and the appearance of the scar. Some women reported feelings of being unable to look at their scar and feelings of shame.

Liz: “Because it has been withdrawn in a certain way [..]” “What do you think about that?” “When I look in the mirror? I think that’s ugly! Yes, it doesn’t look good.” “What do you see?” “Yes, if I look in the mirror ... Now I’m starting to cry... Then I only look at the scar. It’s ugly.”

Important in the perception of scarring is the relation with surviving breast cancer and dealing with their changed body. For some women, scarring was a physical reminder of surviving breast cancer.

Leah: “Well, it’s a part of my life now. But there it is, there is always some sort of raw sadness and it is so long ago! But that still remains…” “Are you mainly talking about the loss of your breast?” “Yes, both actually, but also the loss of mobility. I’m used to doing a lot, I was very agile in my shoulders. And now I’m not anymore, I think that is also restricting.”

In addition, some women mentioned fear of pain and tearing of their scar.

Susan: “Only if you try to grab something and then you feel something tearing in such a way: then you are actually tempted to let everything fall out of your hands. Uhm, that restricts me. It’s also a part of fear, because that pain is just not pleasant.”

#### *Social functioning*

Social impact of scarring was for some women caused by pain and movement restrictions, feeling unable to carry out a hobby or a favorite sport. Visibility of the scar and the need to cover it up were factors reflected in daily life, like going to parties.

Liz: “When I make certain movements, it pulls. The fact that I’m limited in my actions, which I did not have before... for example when I went for a swim... and that’s actually also the case with skiing […].

What makes you so emotional? That I did not have it before…that you could still do all those things... it’s limiting.”

The scars can have impact on self-confidence to maintain and form new (sexual) relationships. Although some women find it difficult to show or discuss their scars to others (partner, family, friends, health-care professionals), others do not.

Lily: “I don’t have problems with someone else looking at it. I will also walk into the sauna…they can look if they want to.” “Have you ever been to the sauna?” “Yes... and yes I didn’t pay attention, I don’t care! *laughter*.”

#### *Cognitive functioning*

Scars seem to have an impact on cognitive functioning for some women. Specifically in being constantly aware of the physical aspects of scarring, like pain and movement restrictions, but moreover the visibility of scars and often trying to hide the scars.

Grace: “It’s not that I suffer from it all day, but you do have daily reminders. Yes, that it’s there: then I feel it or it feels very tight.. and of course the cramps: the cramp I find very annoying. I really don’t like it, because it actually does hurt quite a lot.”

Liz: “So I am very conscious when I go somewhere or have a party or dinner... what kind of clothes to wear? And how high? A shirt underneath? So it’s always something you have to think about. Which I didn’t before. I used to see something on the hanger and think, yes, that’s what I’ll wear, but that’s no longer the case.” “To what extent do you suffer from that?” “That I think about it all day long? Well sometimes it determines the majority of my day.”

### *Environmental factors*

The degree of social support women experience influences the acceptance of their scar, most women expressing the positive influence of social support, especially from their partner. Although a few women did not feel supported by their partner.

Lisa: “It makes a difference that I have a partner who is also very…that is also important: that he also deals with it naturally.”

Seasonal changes were mentioned in relation to provoking pain, stiffness and uncomfortable sensations. Warm weather also influences the possibility to cover up visible scars by clothes.

Emily: “If it’s colder [...] then it really affects my scar, a little like muscle pain plus.”

Emma: “I think it’s really awful, in the winter I don’t mind so much, then I can wear clothes to cover up. [...] Wearing a t-shirt in the summer: I think it’s really terrible.”

#### Theme 3: Personal factors

Other factors mentioned that seem to influence the degree of experienced symptoms are early complications and an overload in activity during wound healing. Sensory and physical scar characteristics, especially visibility, pain, and movement restrictions seem to impact the degree of acceptance of scarring.

Maggy: “It may sound crazy but because it falls so neatly under my clothes, I don’t really mind it.”

Some women desired scar revision for the same scar characteristics.

Susan: “It just has to be released *talking about adherence*. I would really be so happy. I’m just looking forward to the operation. Because then I might be able to move properly again [...] than I’m no longer dependent on someone else.”

Surviving breast cancer or going through (prophylactic) surgery affects the experience of scarring. Women also related scar symptoms to other complications of breast surgery or breast cancer therapy, since women experienced lymphedema, radiation damage, and nerve pain. Most of the women said to have accepted the inevitability of scarring, but others did not. Expectations of scarring, (dis)satisfaction of care, and information provision, age, priority of the scar, change over time, and coping strategies seem to influence level of acceptance.

Evelyn: “Yes I’m scarred for life and I have to deal with it. I can be down in the dumps, but you shouldn’t do that. Yes, I have scars: that’s a part of it.”

Betty: “You just have to learn to accept that it looks like this. That it hurts. And that’s hard for me. I have a hard time accepting things. That’s very difficult.”

#### Theme 4: Impact of scar interventions

Scar interventions mentioned by the women particularly were manual or mechanical soft tissue mobilization, but also silicone products, lipofilling, and steroid injections.

Participants described positive impact of the scar interventions on both sensory and physical scar symptoms, in particular the reduction of the scar adhesion, which resulted in less pain and improvement in mobility. However, women also reported negative impact of the scar interventions like a stretched scar caused by steroid injections, but especially pain and bruising after soft tissue mobilizations. Some women reported a negative impact of going to therapy in everyday life.

Rosie: “I find it tiring. It’s for the best, but I find it tiring, I would rather do something fun, but I know it’s good.”

With regard to self-management of their scar(s) women reported to apply lotion, massage their scar(s), and do exercises, while others reported that they did not know what to do themselves to improve their scar(s), and stated that they missed (specific) information. In general, women reported that they would have liked to be informed more proactively about the scars, what to expect, and possibilities to reduce scar complaints. This was a one of the reasons for dissatisfaction with the provided care.

Suzy: “No. No never. They never tell you anything about the scars. At least not to me!”

Maggy: “Yes, they did, but not how to massage them. They told me to keep it moisturized and to massage, but how to do this? I really have no idea. I was never told how to do that. I just make it up?”

#### Theme 5: Change over time

Change over time was seen in all previous themes. The most apparent change over time that can be seen in sensory symptoms was pain reduction.

Rosie: “In the beginning I had a lot of trouble. Yes! Maybe even more […] the pain was more extreme, yes worse, certainly just after surgery.”

Other changes in sensory symptoms were less sensitivity and uncomfortable sensations. Women experienced a positive influence of time on physical characteristics of the scars in terms of thickness, color, and general appearance. Tight sensations and adherence both improved and got worse over time, accompanied by physical function restrictions. Women discussed change over time regarding emotional impact of the scar(s). For most women scarring was not a priority at first because of surviving breast cancer, for some of them it became more important over time, while for others the acceptance grew with time.

Liz: “I must honestly say that I’m at some point now, that there is a certain acceptance. Which I didn’t have before. Now there’s a certain acceptance because it’s been a few years, it’s just part of me now.”

## Discussion

Through semi-structured interviews with 26 women after prophylactic, oncologic, and reconstructive breast surgery, we found that women seem to suffer of a wider range of breast scar characteristics and symptoms in comparison with previously identified literature [13]. Breast scar characteristics and symptoms impact a person’s physical, social, emotional, and cognitive functioning, thereby affecting HRQOL, depending on several factors, like personal and environmental factors.

Firstly, the present study emphasizes that women after breast surgery can experience a variety of scar characteristics and symptoms to various degrees, determining the impact on HRQOL. So far, merely the impact of breast scar pain has been identified in the literature, affecting the outcome satisfaction [12], awareness, and physical functioning [18]. Our results demonstrate that not only scar related pain but also adherence and accompanied symptoms like stiffness are symptoms that affect physical functioning, which in turn influences social, emotional, and cognitive functioning. Additionally, women in this study reported that a variety of scar characteristics such as visibility, location, texture, and size determined satisfaction with scar appearance. These scar characteristics were also found in donor site scarring after reconstructive surgery impacting satisfaction [11]. Moreover, women mentioned the influence of their breast scar on the appearance of their breast(s), which in some women even caused the feeling of being mutilated, impacting emotional functioning, and influencing social and cognitive functioning. Our findings correspond with previous data that 10–30% of the women are dissatisfied with the appearance of the scar [9, 10] and 18% experienced their scar to be worse than expected [8]. Nevertheless, it contrasts the statement that scarring is not a major cause of concern for women following breast surgery [8].

These new insights underline the complexity and importance of knowledge about scars after breast surgery. Considering scarring as a common late effect after breast surgery, impacting HRQOL, is an important implication. When using patient-reported outcome (PRO) instruments for clinical practice or future research it is important to notice that current breast surgery PRO instruments do not include specific items regarding scarring [19, 20]. Using a validated scar scale, measuring not only physical scar characteristics and symptoms but also the satisfaction with appearance and the impact of scarring is highly recommended. Moreover, these new insights into scar characteristics and symptoms could provide input for developing evidence-based scar interventions in order to improve the HRQOL of women after breast surgery.

Secondly, our study shows a variation in the degree of scars impacting HRQOL among women. The impact of scarring on HRQOL depends on environmental factors, such as social support and personal factors, of which the degree of acceptance is essential. A number of the women said to have accepted the inevitability of scarring as a result of surgery, while others did not and described their scars in a more negative manner. It is plausible that the degree of acceptance is related to the patient’s expectation of the scar(s). A recurring topic of conversation was that women reported the lack of information about scarring resulting from breast surgery. The importance of expectations with regard to breast scarring after breast reconstruction was previously described [21]. It is remarkable that in the recent study of Matthews et al. [21] nearly all women perceived their scars in a positive manner and most women were able to accept their scars [21]. This can be explained by the fact that this study strictly focused on women after breast reconstruction and the (cosmetic) outcome after reconstruction is a more important topic of information in comparison with oncologic breast surgery. These results confirm the importance of sufficient patient information provision in clinical practice to ensure that women have realistic expectations [11, 21] whilst allowing them to make informed decisions [22]. In general, there is a positive relation between appropriate information provision and mental and global HRQOL [23].

Finally, the variation in the degree of scars impacting HRQOL between women can possibly be explained because of women putting their scar in perspective, and reacting differently to their visibly changed body [18]. Although this is not within the scope of this study, we found that the impact of scarring after breast surgery is influenced by the impact of being diagnosed with breast cancer or going through (prophylactic) breast surgery, as also found in previous literature [18]. Other factors possibly interfering with the experienced scar characteristics and symptoms are other perceived complications in the breast area, like lymphedema, radiation damage, and nerve pain, which emphasizes the complexity of scarring after oncologic breast surgery in clinical practice and research. Future exploration of the impact of breast cancer therapy or (prophylactic) surgery on the perception of scarring both physically (the influence of other breast complaints) and emotionally (the impact of going through breast cancer or surgery) is relevant, in order to gain a better understanding of the perception of scarring after breast surgery.

### Limitations

Limitations of this study should be noted. Although the sample was relatively diverse in terms of age, type, and time since surgery and adjuvant therapy, in order to get a broad diversity in scar characteristics and symptoms, the variety of scar experiences may have not been fully explored in the context of these patient characteristics. This could affect the transferability of the study to a specific breast surgery population. Furthermore, most women (21 out of 26) were recruited through dermal therapy practices (primary care), who actively sought therapy for ongoing side effects (mainly lymphedema) after breast cancer treatment. Consequently, this population, which is over-represented in this study, may experiences the impact of breast scars differently, and therefore, the results may not be transferred to all women after breast surgery. It is recommended that new insights from patients’ perspective should be further investigated, in a population after a specific type of breast surgery, recruited in a population that reflects the actual population in order to generalize outcomes in the future research.

## Conclusion

Women after prophylactic, oncologic, and reconstructive breast surgery can experience a diversity and a combination of scar characteristics and symptoms, which play a central role in the perceived impact on HRQOL. This strongly indicates that scarring is an important late effect after breast surgery and should be given more attention in clinical practice and research. Above all, it is crucial to look beyond the symptoms and characteristics of scars after breast surgery, since scarring can have a considerable impact on HRQOL by influencing a person’s physical-, social-, emotional-, and cognitive functioning.

## Data Availability

The code manual including definitions, code rules, and anchor codes, generated during the current study is available from the corresponding author on reasonable request.

## Appendix: Code Manual Summary

**Table:**

Themes	Definitions	Codes
**Theme 1: Sensory and physical symptoms**		
Physical	All experienced physical scar features.	Overall appearance positive or negative, color, width, thickness, location, visibility, size, dog ears, impact of scar on breast appearance, appearance no longer as before, metaphor, accumulating scar tissue, vascularity, shape. Burden, tightness, adherence, retraction, stiffness, and ROM.
Sensory	All scar symptoms that are a result of a disturbed sensation.	Feeling of tearing, sensitivity, numbness, itch, cramp, uncomfortable sensations (tingling, stinging, pinching, stabbing), pain, and tight sensation.
**Theme 2: Impact of scar symptoms**		
Physical functioning	Descriptions of changes due to the scar on physical function.	Moving, activities of daily living; housekeeping, sleep, sports, lifting, leisure, and work.
Emotional functioning	Personal feelings about the scar; how bothered participants were by the scar or scar interventions.	Personal feelings about the scar: how bothered participants were by the scar or scar interventions, e.g., fear of pain, fear of tearing, fear of tumor, anger, feeling of failure, feeling of shame, feeling of loss, relation with breast cancer, unable to look at scar, sadness, and dealing with changed body.
Cognitive functioning	Impact of the scar symptoms on concentration/attention.	Impact of scar symptoms on consciously aware of scars.
Social functioning	Impact due to presence of the scar or scar interventions on social function.	Impact due to presence of the scar or scar interventions on: daily life, going to parties or sauna, openness, relationships, and sexuality.
Environmental factors	External factors that can have a positive or negative impact on the scar or tolerance of the intervention.	Social support and climate changes.
**Theme 3: Personal factors**		
Personal factors-other	Particular background of the individual or individual approach that affects the impact of the scar or scar interventions.	Acceptance of the way things are, acceptance of scars. Coping in relation to age, price you pay in relation to breast cancer, covering of scars, and scar visibility. Desire to change the scar, emotions breast cancer related?, factors affecting scar symptoms, donor site scar, pre-morbid factors, and priority scar. Satisfaction of care, expectations regarding scarring, and early complication.
Personal factors-quality of life	Scar related comments on the things that matter/are meaningful to the individual that have impact on quality of life.	Quality of life (double coded with other factors when comments made on the things that are meaningful to the individual).
**Theme 4: Impact of the scar interventions**		
Impact scar interventions	Changes to the scars, daily routine and adverse effects as a result of scar interventions.	Impact therapy on scar, impact therapy on routine, adverse effects of therapy, referral scar therapy, therapy after breast cancer other than scar, satisfaction scar therapy, self-management, education, type of therapy, plastic surgery, physical therapy, and negotiability of scar with healthcare professional.
**Theme 5: Change over time**		
Scar related change over time	Changes to the scar, attitude or emotion due to the passing of time.	Changes in sensory symptoms (pain, sensitivity, uncomfortable sensations, tight sensations), changes in physical symptoms (thickness, color, overall appearance positive or negative, tightness), changes in physical functioning, changes in emotional functioning, and changes in acceptance of scar.

Five themes according Simons et al. (2016) with formulated definitions and deductively and inductively formulated codes
